# Inhibitory effects of quail egg on mast cells degranulation by suppressing PAR2-mediated MAPK and NF-kB activation

**DOI:** 10.29219/fnr.v62.1084

**Published:** 2018-07-20

**Authors:** Priscilia Lianto, Fredrick O. Ogutu, Yani Zhang, Feng He, Huilian Che

**Affiliations:** 1 Beijing Advanced Innovation Center for Food Nutrition and Human Health, College of Food Science and Nutritional Engineering, China Agricultural University, Beijing, 100083, P.R. China; 2 College of Food Science and Nutritional Engineering, China Agricultural University, Beijing, 100083, P.R. China; and; 3 Food Technology Division of Kenya Industrial Research and Development Institute, South C – Popo Rd., Off Mombasa Rd., PO Box 30650-00100, Nairobi, Kenya

**Keywords:** quail egg, mast cells, anti-allergic, PAR-2, degranulation, activation

## Abstract

**Background:**

Quail egg (QE) has been reported to possess an anti-allergic and anti-inflammatory activity. We have demonstrated that whole QE was able to attenuate the allergic symptoms in food allergy–induced EoE murine model, but whether QE albumen or QE yolk plays a more important role still remains unclear.

**Objective:**

In this current study, we investigated the suppressive role of QE in mast cell degranulation and cytokine production of the effect phase response.

**Method:**

A passive cutaneous anaphylaxis (PCA) mouse model was used to confirm the anti-allergic effect of QE. Besides, HMC-1 cell model was used to study its suppressive role in more detail. In this *in vitro* study, we divided QE into three groups: whole QE, QE albumen, and QE yolk. The effect of QE treatment on mast cell degranulation and intracellular calcium influx was investigated. Moreover, the effect of QE allergy– related mediators, genes, and proteins were also assessed by ELISA, RT-PCR, and western blotting.

**Results and discussion:**

Our data showed that the extent of mast cell degranulation–mediated ear vascular permeability in IgE-mediated PCA mice treated with whole QE (17 mg/kg) was decreased significantly up to 43.31 ± 0.42% reduction. HMC-1 cell–based immunological assay in vitro indicated that QE, particularly its albumen, acted as a ‘mast cell stabilizer’. Under the concentration of 70 μg/mL, QE albumen effectively suppressed the releases of β-hexosaminidase, histamine, and tryptase, as well as Th2 and pro-inflammatory cytokine production; reached 30 up to 50% reduction. Besides, QE albumen was also able to significantly modulate the upregulation of IL-10 up to 58.30 ± 5.9%. Interestingly, our data indicated that QE yolk still had a significant inhibitory effect on modulating Th2 cytokines in its highest concentration (100 μg/mL), while QE albumen showed no inhibitory effect. Western blot analysis showed QE albumen effectively down-regulated the expressions of calcium-related protein (TRPC1, Orai1, STIM1, PLC-γ and IP3R), facilitated the reduction of PAR-2 and induced the reduction of phosphorylation of JNK, IKKα, p50 and p65 protein expressions.

**Conclusion:**

As confirmed by PCA and HMC-1 cell-based immunology assay, QE albumen and QE yolk may work together through exerting anti-allergy activity and can be used as a potential anti-allergic nutrient in the future.

Pervasiveness of allergic diseases such as atopic dermatitis, asthma, food allergy, hives, and hay fever is a serious health issue. These diseases are classified into type I hypersensitivity caused by release of the granule-stored mediators such as histamine, proteases, lipid mediators, and cytokines from mast cells (1). In addition, mast-cell mediators apparently through the protease-activated receptor (PAR)-2 may likewise contribute to the development of unending and allergic inflammation by supporting the inflow of inflammatory cells, such as eosinophils, macrophages, lymphocytes and basophils, prompting tissue inflammation (2). Thus far, there are no treatments accessible to cure allergic diseases entirely. However, a few pharmaceuticals as well as mast cell stabilizers (disodium cromoglycate, sodium hydroxypropylcromate, ketotifen, etc.), anti-histamine drugs (diphenhydramine, chlorpheniramine maleate, terfenadine, etc.), and immune suppressors (adrenal cortical hormones, dexamethasone, hydrocortisone, etc.) are essentially utilized to help relieve unfavorable allergic symptoms and reduce the distress of anaphylaxis through stabilizing mast cells for treating allergic disease. When mast cells are stabilized, they are not readily triggered by stimulatory factors, such as allergens, to engage in steps leading to the discharge of preformed pharmacologic mediators and the new synthesis of inflammatory lipid mediators and cytokines (3). However, these medications not only have side effects but also do not counteract symptom reoccurring. Therefore, natural anti-allergic ingredients would be a suitable option for anti-allergic strategy.

Previous studies suggest that quail egg (QE) is different from other bird eggs. This distinction particularly originates from its egg albumen, which is richer in proteins that have anti-allergic and anti-inflammatory effects (4–6). A patent on the role of QE in controlling the immune cell function, particularly eosinophils and neutrophils, in the treatment of hypersensitivity, was issued in 2015 by the United States (patent number: US2015/0057232A1) (7), giving strong establishments of the unique role of QE. A number of clinical studies have also indicated the therapeutic effect of QE as allergic asthmatic (8) and rhinitis treatments (9). More recently, our study has also shown the therapeutic potential of QE in in attenuating the symptoms of murine model food allergy induced Eosinophilic esophagitis like disease (10). However, the suppressive role by which QE inhibits mast cell degranulation and allergic type I responses is not well defined.

In this study, we investigated the suppressive effect of QE on modulating mast cell activation–mediated immediate allergic response. Herein, we used PCA and HMC-1 *in vivo* and *in vivo* mast cell model experiments to describe suppressive effects of QE on modulating mast cell degranulation–mediated immediate allergy hypersensitivity.

## Materials and methods

### Chemicals

The chemicals were obtained from the following suppliers: monoclonal anti-Dinitrophenyl antibody produced in mouse (anti-DNP IgE, D8406), dinitrophenol-human serum albumin (DNP-HSA), Compound 48/80 (C48/80, C2313), water-soluble tetrazolium-8 (WST-8, 96992), 4-nitrophenyl N-acetyl-b-D-glucosaminide (N9376), Evans blue (E2129), and Fluo-3AM (39294) (Sigma–Aldrich Corp., USA); TransCript One-Step gDNA Removal and cDNA Synthesis SuperMix (AT311) and TransStrart Top Green qPCR SuperMix (AQ131) (TransGen Biotech, China); Commercial human ELISA kits (eBioscience, Inc., San Diego, CA); BCA Protein Assay Kit (CW0014S, CWBiotech, Beijing China). Anti-β-actin antibody (ab36861), anti-Transient Receptor Potential Channel 1 (TRPC-1) antibody (ab192031), anti-Calcium Release Activated Calcium Channel Protein 1 (Orai1) antibody (ab83751), anti-Stromal Interaction Molecule 1(STIM1) antibody (ab59342), anti-*Phospholipase C-gamma (*PLC-γ) antibody (ab37384), anti- and Inositol 1, 4,5-Trisphosphate Receptor (IP3R) antibody (ab38557), anti-Extracellular Signal-Regulated Kinase (ERK1/2) antibody (ab30258), anti-p38 antibody (ab30359), anti c-Jun N-Terminal Kinase (JNK1/2) antibody (ab303154) (Abcam, UK); anti-IkappaBalpha (IKK-α) antibody (397700), anti-Nuclear Factor Kappa B (NF-kB) p50 antibody (14-6732-81), anti NF-kB p65 antibody (14-6731-63), (eBioscience, Inc., San Diego, CA). All other reagents used in this study were of analytical grade.

### Sample preparation

Normal, commercially available, fresh QE (*Coturnix* sp.) were obtained from a local egg market. QE were divided into three groups: whole QE (egg albumen and yolk), QE albumen (albumen separated from egg yolk), and QE yolk (yolk separated from egg albumen). Each of the groups was mixed using mixer (Joyoung Co Ltd.), freeze-dried and powdered using vacuum freezer (Alpha 1-2 LD plus, Martin Christ, Germany), and then packed and stored at −20°C.

### Animals and management

Female BALB/c mice aged 7–8 weeks were purchased from Weitong Lihua Experimental Animal Technology Co., Ltd. (Beijing, China; No: SCXK(Jing)2016-0001) and acclimatized to their new housing for a week before beginning experimental protocols. Animal experiments employed age-, gender-, and genetic-strain-matched controls to account for any variations in data sets compared across experiments. Mice were bred and housed under specific pathogen-free (SPF) conditions in the animal laboratory of College of Food Science and Nutritional Engineering, China Agricultural University (Beijing, China). Experimental mice rooms were maintained at a temperature of 23 ± 3°C, relative humidity of 40–70%, light/dark cycle of 12 h, and air exchanges at 15 times/hour. Experimental mice were provided with *ad libitum* access to fresh filtered water and standard rodent diet (moisture, ash, crude protein, fat, crude fiber, calcium, and phosphorus) produced by Ke Ao Xie Li Feed Co., Ltd. (Beijing, China). It met Chinese Standard GB14924.3-2010 feeding condition requirement, and the limit of detection for aflatoxin was below 20 μg/kg. All experiments were performed under the China Agricultural University Animal Experimental Welfare and Ethical Inspection Committee approved protocols and in accordance with its guidelines. All efforts were made to minimize the suffering of experimental animals.

### Establishment of IgE-mediated PCA model in BALB/c mice

The PCA assay was carried out following procedure previously described by Knoops et al. (11). Basically, one ear of the animal is injected with anti-DNP-IgE, and the other is left alone. Thus, 1 h post-dosing animals are challenged with a tail vein injection of DNP-HSA diluted in Evan’s blue dye. Signs of blue reaction and swelling from the site of IgE-sensitized ear represent mast cell degranulation. One hour after the last injection, mice are euthanized and ear punch biopsies from both ears are harvested to quantify Evan’s blue content in the ear tissue using spectrophotometric techniques for measuring dye extravasation into the tissue. The PCA assay in this study was performed according to the schedule set-forth in this study protocol with slight modifications.

Ten female BALB/c mice (4 weeks old, weighing 18–22 g) were divided into two groups (*n* = 5). All tested mice received an intradermal injection of 0.5 μg of anti-DNP IgE in 30 μL of saline in the right ear. A week prior to sensitization, BALB/c mice were given 17 mg/kg∙bw of daily oral egg treatments. The amount for oral administration was followed by the recommended dietary intake of QE by Integrative Therapeutics (integrativepro.com/allqlear • 800.931.1709) which is 84 mg/day for humans (12). For the dose conversion of QE dietary treatment, we followed a Simple Practice Guide for Dose Conversion between Animals and Human by using the average weight of human body in China, which is around 60 kg (13). The formula of calculation is:

$$\text{Human}\;\text{equivalent}\;\text{dose}\;\text{(mg/kg)=mouse}\;\text{equivalent}\;\text{dose}\;\text{(mg/kg)×}\frac{\text{dosage}\;\text{conversion}\;\text{factors}\;\text{for}\;\text{mouse}}{\text{dosage}\;\text{conversion}\;\text{factors}\;\text{for}\;\text{human}}\frac{\text{84mg}}{\text{60mg}}=\text{mouse}\;\text{equivalent}\;\text{does}\;\text{(mg/kg)×}\frac{3}{37}$$

Therefore, in this experiment, the dose of QE was 17 mg/kg weight body/animal. During the experiment period, QE-treated BALB/c mice were also given concurrently continuous access to QE by feeding water containing 17 mg/L QE. On day 8, each mouse was injected intraperitoneally with 200 μL of DNP-HSA and Evans blue solution (100 μg DNP-HSA and 2% Evans blue in 0.9% NaCl). After challenge, Evans blue extravasation in the right ear was captured by a Canon Electro Optical System (EOS) camera (Canon, Inc., Japan) as a qualitative analysis of vascular permeability. The mice were sacrificed 40 min after treatment with DNP-HSA, and right ears were collected. The ear dye color was extracted by incubating ear with formamide at 64°C for 12 h. The absorbance of the dye was determined at 620 nm using the Thermo Scientific Varioskan Flash (Thermo, USA) to quantitatively assess the vascular permeability.

### HMC-1-cells-based immunological assay

The human mast cell line (HMC-1) obtained from national platform of experimental cell resources (Beijing, China) was cultured in RPMI 1640 medium supplemented with 10% fetal bovine serum (FBS) and 1×10^5^ U/L penicillin/streptomycin at 37°C in a humidified 5% CO_2_ incubator. Thus, this study using HMC-1 mast cell line conducted mast cell viability and degranulation experiment assays.

Cell numbers of HMC-1 after QE pre-treatments were examined using WST-8 assay kit according to manufacturer’s instructions. WST-8 is reduced by dehydrogenases in cells to give an orange-colored product (formazan), which is soluble in the tissue culture medium. The amount of the formazan dye generated by dehydrogenases in cells is directly proportional to the number of living cells (14). In brief, 1×10^4^ HMC-1 cells/mL medium were pre-incubated with various concentrations of 0, 50, 100, 500, and 1000 μg/mL of QE groups. After incubation for 30 min, 10 μL of WST-8 was added for incubating for another 30 min at 37°C. Finally, supernatants were transferred into another 96-well plate for measurement at 450 nm with a Thermo Scientific Varioskan Flash plate reader (Thermo, USA).

The HMC-1 cell–based immunological assay was carried out following procedure previously described by Hohman & Dreskin with some modification (15). Firstly, the cells were pre-incubated with QE group treatments with various concentrations (0, 50, 70, 100 μg/mL) for 30 min. Afterward, the cells were stimulated with 100 μg/mL C48/80 for 45 min at 37°C. After stimulation with C48/80 for 1 h, cell solution was centrifuged at 1,500 rpm for 5 min, 30 μL of supernatant was collected and transferred to a 96-well plate and incubated with 50 μL of p-Nitrophenyl-N-Acetyl-β-D-Glucosaminide (1.3 mg/mL in 0.1 M citric acid buffer, pH 4.5) for 1 h at 37°C. The reaction was terminated by adding 200 μL stop solution (0.1 M Na_2_CO_3_/NaHCO_3_, pH 10.0). Each well absorbance was measured at 405 nm using a Thermo Scientific Varioskan Flash microtiter plate reader (Thermo, USA). The total release of β-hexosaminidase was determined in HMC-1 cells without egg treatments and the spontaneous release of HMC-1 cells was evaluated by adding 50 μL of medium only instead of C48/80 to each well. Aliquots of the cell lysate or culture supernatant were sonicated in 130 μL of the modified medium containing 0.1% Triton X-100 at 37^°^C for 45 min. The release of β-hexosaminidase activity was measured using 4-nitrophenyl 2-acetamido-2-deoxy-β-D-glucopyranoside (PNAG) as described previously by Kuehn et al. (16). The formula of the release of β-hexosaminidase calculation is:

$$\text{β-hexosaminidase release }\left(\text{100\%}\right)=\frac{{\mathrm{Absorbance}}_{\mathrm{supernatant}}{-\mathrm{Absorbance}}_{\mathrm{blank}\;\mathrm{of}\;\mathrm{supernatant}}}{{\mathrm{Absorbance}}_{\mathrm{total}\;\mathrm{release}}{-\mathrm{Absorbance}}_{\mathrm{cell}\;\mathrm{lysate}}}\text{×100\%}$$

### Measurement of allergic mediators and cytokines

The HMC-1-based immunological assay on detecting histamine and tryptase release levels was performed similar to the β-hexosaminidase release detection assay, while for the detection of Th2 cytokines (IL-4, IL-5, IL-10 and IL-13) and pro-inflammatory cytokines (IL-6, IL-8, and TNF-α), this study conducted a slight modification of cell stimulation time. After QE-pretreated HMC-1 cells were stimulated with C48/80 for either 1 h or 8 h, cell solution was centrifuged at 1,500 rpm for 5 min. Culture supernatants of cells were collected and stored in −80^°^C prior to mediator and cytokine level analysis. All these allergic mediators and cytokines were determined using ELISA kit according to the manufacturer’s instructions (eBioscience, Inc., San Diego, CA).

In brief, each mediator standard was set and 50 μL standard diluent was added to standard well. 10 μL testing sample was added to testing sample well, and then was followed by addition of 40 μL sample diluent. Blank wells were not added anything. 100 μL of horseradish peroxidase-conjugated streptavidin (HRP labelled avidin working fluid) was added to each well, covered with an adhesive strip, and incubated at 37^°^C for 60 min. Each well was aspirated and washed five times with phosphate-buffered saline (pH 7.4) containing 0.1% Tween-20 (PBST washing dilution). 50 μL chromogen solution A and 50 μL chromogen solution B were added, gently mixed, and incubated at 37^°^C for 15 min with no light condition. In the end, 50 μL terminate solution (100 μL of 2 N sulfuric acid) was added to each well, gently mixed, and then within 5 min, the absorbance value of each hole was measured at 450 nm wavelength using a microplate reader Thermo Scientific Varioskan Flash (Thermo, USA). The detection limits of allergic mediators and cytokines were as follows: histamine (0.1 ng/mL), tryptase (1.0 ng/mL), IL-5 (0.1 pg/mL), IL-4, IL-6, IL-10, IL-13, (1.0 pg/mL), TNF-α (1.7 pg/mL), and IL-8 ECP (2.0 pg/mL).

### Measurement of intracellular calcium (Ca^2+^) concentration

Intracellular calcium influx was measured according to the previous method described by Huber et al. (17). Cells were seeded into a 96-well black opaque cell culture plate. After pre-incubation with or without QE treatments, cells were incubated with 5 μM of Fluo-3AM for 30 min at 37°C in the dark condition. Following a 30s baseline recording, cells were challenged for another 300s with 100 μg/mL of C48/80. The fluorescence intensity (FI) was recorded using the Thermo Scientific Varioskan Flash microtiter plate reader (Thermo, USA) with excitation wavelength at 488 nm and emission wavelength at 525 nm. The formula of [Ca^2+^]_i_ calculation is:

[Ca^2+^]_I_ (nm) = K_d_[(F-F_min_)/(F_max_-F)]

where F_min_ is the background fluorescence with 5 mM EGTA and F_max_ is the maximum fluorescence with 0.1% Triton X-100 instead of C48/80. The effective dissociation constant (Kd) of Fluo-3 and Ca^2+^ is 400 nM.

### RT-PCR analysis

QE-pretreated HMC-1 cells were stimulated with C48/80 for 6 h. Total RNA was extracted using Trizol reagent and cDNA was transcribed using the TransScript One-Step gDNA Removal and cDNA Synthesis SuperMix. RT-PCR was conducted using the TransStart Top Green qPCR SuperMix for PAR-2 and β-actin. PCR for HMC-1 was performed with primers as follows:

5’-TTTCTCTCGGTGCGTCCAG-3‘ (sense) and

5’-GTTCCTTGGATGGTGCCACT-3‘ (anti-sense) for PAR2;

5’-CTCGCCTTTGCCGATCC-3’ (sense) and 5’-GGGGTACTTCAGGGTGAGGA -3’ (anti-sense) for β-actin.

Quantitative real-time PCR conducted under the thermal cycling conditions involved denaturation step at 95°C for 1 min, followed by 40 cycles at 94°C for 30s, 60°C for 30s, 72°C for 30s, and the final melting curve program with raping rate 0.5°C/0.05 sec from 55°C to 95°C. Using β-actin as the internal control gene, the relative quantitative level of mRNA was calculated by 2^–ΔΔCt^ method C_t_ is the threshold cycle and ΔCt was calculated from test C_t_-β actin C_t_.

### Western blotting

Western blotting was performed according to the previous method described by Mahmood & Yang (18). For the detection of calcium channel–related protein expression, QE-pretreated HMC-1 cells were stimulated with C48/80 for 1 h, while for the detection of mitogen-activated protein kinases (MAPK) and nuclear factor-kappaB (NF-kB) cell signaling pathway–related protein expression, cells were stimulated for 6 h. After stimulation, HMC-1 cell samples were lysed with lysis buffer containing protease inhibitors, and centrifuged at 4°C and 12,000 g for 15 min. The supernatants were collected and the protein concentration of the supernatant was determined using a BCA Protein Assay Kit. The total cell lysates (50–70 μg of total protein) from different samples were subjected to 10% SDS-PAGE, transferred to nitrocellulose membranes blocked in TBST solution containing 5% BSA for 1 h at room temperature. After blocking with 5% BSA in TBST solution, the membrane was, respectively, incubated overnight at 4°C with primary antibodies against β-actin, TRPC1, Orai1, STIM1, PLC-γ, and IP3R, ERK1/2, JNK1/2, NF-kB p50, and p65, (1:1000), or IKK-α (1:400). After washing six times with TBST, the membranes were next probed with HRP-conjugated secondary antibody and incubated for 1 h at room temperature. Protein bands were visualized with enhanced chemiluminescence reagent and exposed to an X-ray film (Sage creation Mnin Chemi II, China).

### Statistical analysis

Statistical analysis was determined by one-way analysis of variance (ANOVA) using GraphPad Prism 5.01 (GraphPad Software, Inc., USA). All data are presented as the mean values ± standard error of the mean (*SEM*) with three times experimental replicates and statistical significance was set at *p* < 0.05.

## Results

### The effect of QE on mast cell activation in IgE-mediated PCA response mice model

**Fig. 1:**
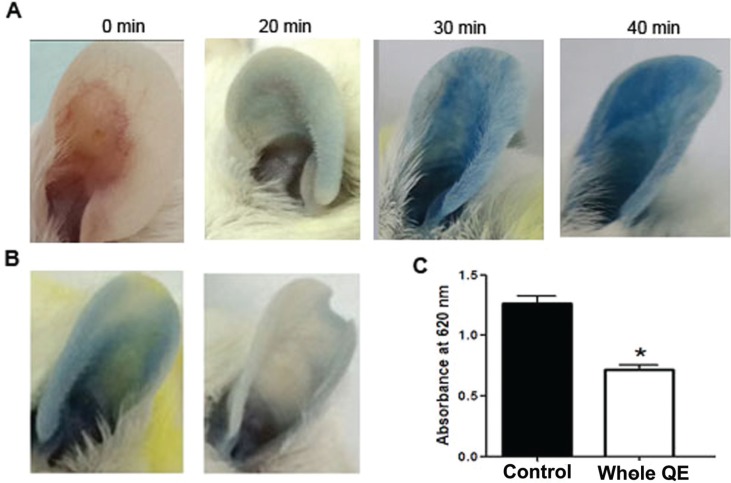
The effects of QE treatment on the vascular permeability and mast cell stabilization *in vivo.* (a) The change of ear color after 40 min DNP-HSA stimulation. (b) Qualitative and (c) quantitative detection of the vascular permeability after Evan’s Blue Dye/DNP-HSA stimulation. Results are expressed as mean ± SEM. *p < 0.05 as compared to the control group.

Type I allergic hypersensitivity is characterized by the abundant activation of mast cells, inducing immediate allergic reactions (19). In order to investigate the role of QE in type I allergic hypersensitivity, the anti-allergic activity of QE *in vivo* was evaluated using IgE-mediated PCA mice. As shown in Fig. 1a, the IgE-mediated PCA was successfully induced by the sequential injections of IgE and DNP-HSA within 40 min after injection. This PCA response was in parallel with vastly occurring capillary dilatation and the increase of ear vascular permeability, by which manifestation was visibly shown by the leakage of Evans blue dye into the ears, indicating the occurrence of mast cell degranulation at site of IgE-sensitized mice ears. When QE was orally administered to IgE-mediated PCA mice, the extent of mast cell degranulation–mediated ear vascular permeability was lessened, as indicated by the intensity of blue color of the ear and Evans blue extraction of the ears. The absorbance of the dye showed that the PCA reaction was significantly suppressed by oral administration as compared to control group (1.27 ± 0.12 vs 0.72 ± 0.09; *p* < 0.05; Fig. 1b).

From the PCA experiment, we might identify the effect of oral QE treatment in inhibiting mast cell degranulation–regulated mice vascular permeability symptom in IgE-mediated PCA mice. The suppressive effect of 17 mg/kg of QE was similar to our previous study using food allergy–induced EoE murine model (10). In order to elucidate the detail suppressive role of how QE modulates mast cell activation, we conducted further experiment using HMC-1 cell line. We divided QE into three groups: whole egg, egg albumen, and egg yolk in order to investigate which parts of QE could act as anti-allergic and inflammatory agents.

### The effect of QE on HMC-1 mast cell activation

**Fig. 2:**
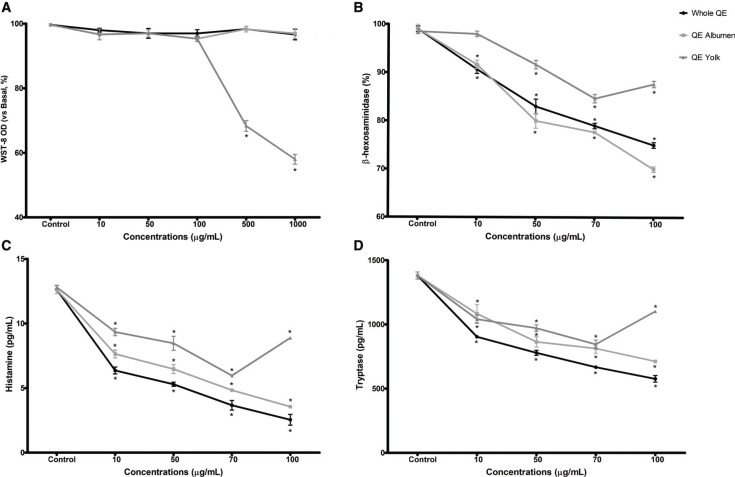
The effects of QE treatments on HMC-1 mast cell stabilization *in vitro.* (a) Cell viability and cell degranulation–mediated mediator release. (b) β-hexosaminidase, (c) histamine, and (d) tryptase levels. Results are expressed as mean ± SEM. *p < 0.05 as compared to control group.

In most cases of using therapeutic compound treatment, it can influence the viability of treated cells (14). We first examined the cytotoxic effect of QE on HMC-1 cells using the WST-8 assay. HMC-1 cells were treated for 30 min at final QE group concentrations of 10, 50, 100, 500, and 1000 μg/mL. The results showed that the viabilities of cells treated with different QE groups were not affected within the treated concentration range, except for cells treated with QE yolk, as shown in Fig. 2a. In addition, we found no significant difference between cells treated with either whole or albumen QE groups and control group (*p* > 0.05). However, we found significant reduction of cells treated with QE yolk at concentrations ranging from 500 to 1000 μg/ mL, which reduced almost 40% from original numbers of cells (1.40 ± 0.05 vs 0.82 ± 0.07; *p* < 0.05). Therefore, we used QE concentrations ranging from 10 to 100 μg/ mL for all subsequent experiments.

Degranulation is an important sign of mast cell activation. In order to carry out a more comprehensive examination of degranulation levels, we investigated not only the level of β-hexosaminidase release but also the levels of histamine and tryptase release which are valid biomarkers for mast cell degranulation (14). The effect of various QE treatments on degranulation of HMC-1 cells was examined. HMC-1 cells were treated with various QE treatments, and degranulation was induced by C48/80. As expected, the release of β-hexosaminidase was significantly increased over the incubation time with C48/80 as shown in Fig. 2b.

Accordingly, C48/80 challenged HMC-1 cells were incubated for 1 h to determine the effect of QE on β-hexosaminidase release. QE suppressed the release of β-hexosaminidase in a dose-dependent manner (Fig. 2b). On the contrary, histamine and tryptase were also robustly released following the C48/80 stimulation (Fig. 2c-d). In line with β-hexosaminidase, QE treatments indicated similar inhibition effect on modulating histamine and tryptase release from HMC-1 cells (Fig. 2c-d). QE suppressed the β-hexosaminidase, histamine, and tryptase released *in vitro* with HMC-1 in a dose-dependent manner, which showed significant effect in the range concentrations of 50–100 μg/mL (*p* < 0.05), reached 30 up to 50% degranulation reduction. Data also indicated that the degranulation suppression of QE albumen was stronger than QE yolk (*p* < 0.05). This reduction difference could be shown by the comparison degranulation reduction levels for β-hexosaminidase, histamine, and tryptase between QE albumen and yolk groups, wherein reductions, under 100 μg/mL QE concentrations, reached 47.03 ± 1.23% vs 29.15 ± 1.96%; 72.41 ± 0.08% vs 30.04 ± 0.42%; and 50.43 ± 1.92% vs 34.99 ± 1.83%, respectively. In addition, cells treated with the highest concentration of QE yolk (100 μg/mL) showed a recovery level of mediator releases wherein reductions were lower than cells treated with 70 μg/mL of QE yolk (β-hexosaminidase: 44.67 ± 1.53; histamine: 5.91 ± 0.15; tryptase: 1109.52 ± 5.52). These results suggest that QE suppressive effect on modulating mast cell degranulation is likely derived from QE albumen.

### The effect of QE on HMC-1-released Th2 cytokines

**Fig. 3:**
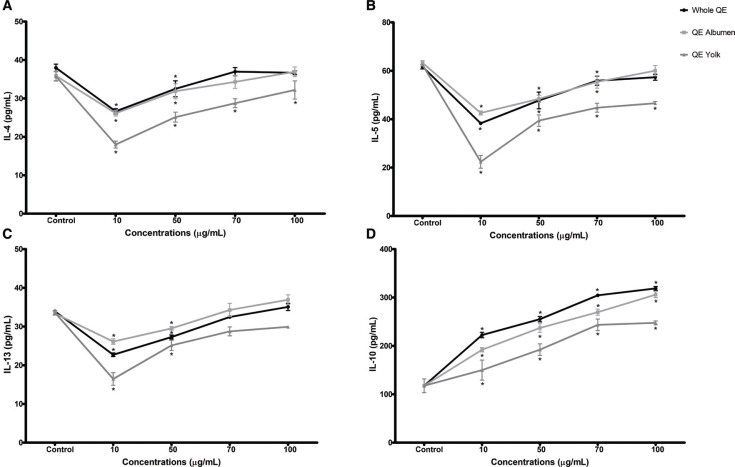
The effect of QE treatments on HMC-1 released Th2 cytokines. (a) IL-4, (b) IL-5, (c) IL-13, and (d)IL-10. Results are expressed as mean ± SEM. *p < 0.05 as compared to the control group.

Mast cells have a vital role as crucial effector and controlling cells in the initiation of the allergic immune response by regulating Th2 lymphocyte differentiation and thus prompting the generation of Th2-associated cytokines (20). We, therefore, investigated the effect of QE on Th2-cytokines (IL-4, IL-5, and IL-13). As shown in Fig. 3a-c, the inhibitory effects of different QE treatments on modulating Th2 cytokines (IL-4, IL-5, and IL-13) showed similar results. As compared to control group, all QE treatments showed inhibitory effects on modulating these Th2 cytokine levels in a reverse dose-dependent manner. The higher the concentrations of QE, the weaker the inhibition effects on modulating Th2 cytokines (*p* < 0.05).

As compared to control group, the significant inhibitory effects of whole QE and QE albumen on modulating IL-4 (whole QE: 31.77 ± 2.53; QE albumen: 30.93 ± 2.47 vs C group: 36.68 ± 2.29) and IL-13 (whole QE: 28.67 ± 2.21; QE albumen: 29.52 ± vs C group: 33.68 ± 0.64) cytokine levels were only up to 50 μg/mL concentration, while the significant inhibitory effects of whole QE and QE albumen on modulating IL-5 cytokine level were up to 70 μg/mL concentration (whole QE: 54.14 ± 3.82; QE albumen: 55.41 ± 3.67 vs C group: 62.50 ± 1.80). Yet, under the highest concentration of QE treatment (100 μg/mL), we found no significant IL-4 (whole QE: 37.78 ± 1.79; QE albumen: 36.21 ± 1.68 vs C group: 36.68 ± 2.29), IL-5 (whole QE: 59.39 ± 3.85; Q albumen: 60.04 ± 3.05 vs C group: 62.50 ± 1.80), and IL-13 (whole QE: 34.58 ± 1.28; QE albumen: 36.38 ± 1.90% vs C group: 33.68 ± 0.64) level differences. In contrast, as compared to control group, QE yolk, up to 100 μg/mL concentration, still showed significant inhibitory effects on the releases of IL-4 (30.09 ± 4.70 vs 36.68 ± 2.29) and IL-5 (46.59 ± 1.01 vs 62.50 ± 1.80) but not in the release of IL-13 (29.84 ± 0.22 vs 33.68 ± 0.64; *p* < 0.05). All of these results indicated that QE yolk was likely the most effective regulator in modulating the release of Th2-associated cytokines.

Mast cell-derived IL-10 restricts leukocyte infiltration, inflammation, tissue damage associated with innate response and thereby preventing damage to the organ (21). As shown by Fig. 3d, all QE treatments showed a similar effect on upregulating IL-10 level of HMC-1 cells in dose-dependent manner. As compared to control group (127.52 ± 4.17), data indicated that treated cells under the highest concentration of whole QE, QE albumen, and QE yolk (100 μg/mL) demonstrated a marked upregulated IL-10 level, reaching up to 315.51 ± 5.86, 305.84 ± 8.81, and 247.56 ± 3.92, respectively (*p* < 0.05). However, QE yolk showed the weakest upregulation effect on modulating the level of IL-10 release, which was almost 20% lower than whole QE or QE albumen IL-10 upregulated levels (*p* < 0.05). This finding indicated that QE albumen was more effective in upregulating the release of IL-10 rather than QE yolk.

### The effect of QE on HMC-1-released pro-inflammatory cytokines

**Fig. 4:**
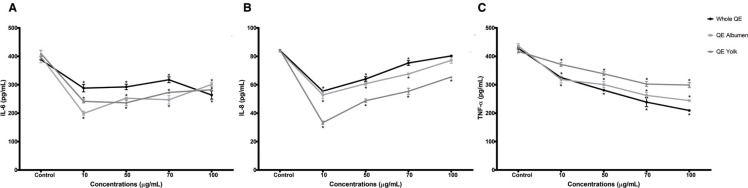
The effect of QE treatments on HMC-1 released pro-inflammatory cytokines. (a) IL-6, (b) IL-8, and (c) TNF-α. Results are expressed as mean ± SEM. *p < 0.05 as compared to the control group.

Mast cell activation also results in the vast release of pro-inflammatory mediators (22), leading to the exacerbation of allergic response. Therefore, we also investigated whether various QE treatments were able to modulate the release of pro-inflammatory cytokines. Hence, we measured the levels of IL-6, IL-8, and TNF-α release from HMC-1, as shown in Fig. 4.

As compared to the control group, QE albumen and QE yolk showed similar significant inhibitory effect on the release of IL-6 in a reverse dose-dependent manner, in the highest concentration of treatment (100 μg/mL), and was able to reach the highest value of IL-6 cytokine release (QE albumen: 301.49 ± 11.45; QE yolk: 282.32 ± 6.12 vs C group: 399.22 ± 17.95; Fig. 4a, *p* < 0.05). In addition, whole QE which conversely showed significant inhibitory effect on the release of IL-6 in a dose-dependent manner, in the highest concentration of treatment (100 μg/mL), was able to reach the lowest value of IL-6 cytokine release (Whole QE: 238.00 ± 11.56 vs C group: 399.22 ± 17.95; Fig. 4a, *p* < 0.05).

Meanwhile, for the release of IL-8 cytokine, all QE treatments showed significant inhibition effect on modulating IL-8 cytokine level in a reverse dose-dependent manner. As compared to control group, cells treated with the highest concentration of whole QE and QE albumen (100 μg/mL) were able to reach the IL-8 level of control group (whole QE: 82.24 ± 3.40; QE albumen: 77.12 ± 2.97 vs C group: 83.86 ± 0.75, Fig. 4b, *p* > 0.05). In contrast, even QE yolk inhibition effect also showed a reverse dose-dependent manner, but the highest concentration of QE yolk still showed significant inhibitory effect on modulating IL-8 level (64.22 ± 2.32 vs 83.86 ± 0.75, Fig. 4b, *p < 0*.05). The highest concentration of QE yolk (100 μg/mL) was only able to lower the release of IL-8 cytokine level up to 23.42 ± 2.11%, whereas the lowest concentration of QE yolk (10 μg/mL) was able to lower the release of IL-8 up to 52.47 ± 3.78% (Fig. 4b, *p* < 0.05), suggesting the higher the concentration of QE yolk, the weaker the inhibition effect on the release of IL-8 level.

In contrast to the other two pro-inflammatory cytokine release patterns, all QE treatments showed a significant inhibitory effect on modulating the release of TNF-α in a dose-dependent manner (Fig. 4c, *p* < 0.05). The highest concentration of QE treatments (100 μg/mL) was able to suppress the release of TNF-α to reach 209.31 ± 4.25; 238.77 ± 2.89; 294.3 ± 3.72 for whole QE, QE albumen, and QE yolk, respectively, as compared to the control group (428.29 ± 13.47). This suppression effect of whole QE, QE albumen, and QE yolk on the release of TNF-α caused a marked reduction up to 51.13 ± 2.17%; 44.25 ± 2.58%; 31.28 ± 2.85%, respectively. Not similar with the IL-6 and IL-8 cytokine modulating trend observed in this study, the inhibition effect of QE albumen on modulating TNF-α was stronger than QE yolk (Fig. 4c, *p <* 0.05). Overall, we concluded that each of the QE treatments likely through differently regulating cell signaling pathways brought different results in modulating pro-inflammatory cytokines.

As most of the cases we found in this study indicated that 70 μg/mL of QE treatments were effective to modulate the inhibition response of HMC-1 cells; therefore, we used this range of concentration of QE treatments for further experiments.

### The effect of QE on the influx of intracellular ion calcium (Ca^2+^) of HMC-1 cells

**Fig. 5:**
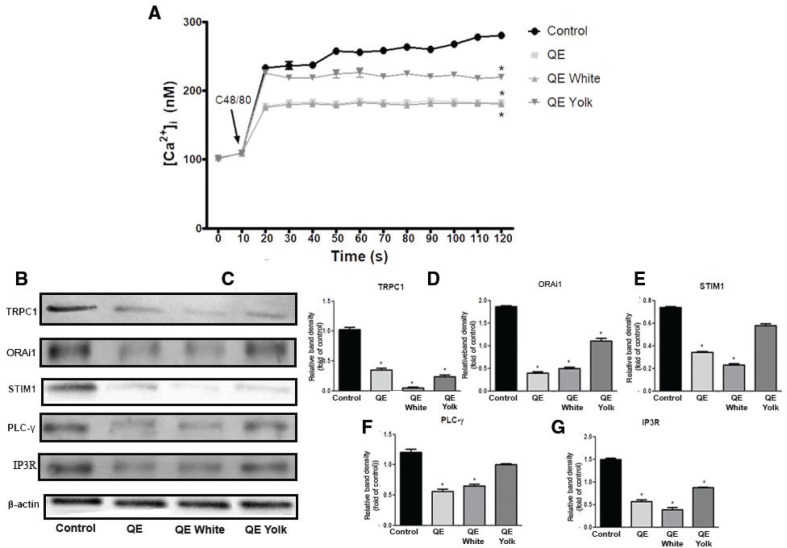
The Effect of QE on the influx of intracellular ion calcium (Ca^2+^) of HMC-1 cells. (a) Intracellular Ca^2+^ influx concentration. (b) Western blotting of calcium protein expression. Fold activation data analysis: (c) TRPC1, (d) ORAi1, (e) STIM1, (f) PLCγ, and (g) IP3R. Results are expressed as mean ± SEM. *p < 0.05 as compared to control group.

As reported, the degranulation of mast cells depends on intracellular calcium ion [Ca^2+^]_i_ release from the endoplasmic reticulum (ER) and calcium release–activated calcium-mediated Ca^2+^ influxes (23). We further investigated the effect of QE treatments on Ca^2+^ influx. Fluo-3AM, a fluorescent Ca^2+^ indicator, was used to determine the [Ca^2+^]_i_. As shown in Fig. 5a, [Ca^2+^]i in the control cells rapidly increased after stimulation with C48/80. Elevation of [Ca^2+^]i in the cells treated with whole QE and albumen QE was significantly suppressed until it reached level 183.31 ± 1.92 and 180.15 ± 0.62, as compared to control group wherein [Ca^2+]^i influx level was still high during 120s up to reaching 279.99 ± 0.82 (*p* < 0.05), and kept its gradual progression. In addition, QE yolk also showed suppressive effects on [Ca^2+^]_i_ even though its suppressive effect was weaker than other QE treatments, by which it reached only 219.18 ± 0.54 (*p* < 0.05). These results indicated that QE, particularly its albumin, was effective in inhibiting the elevation of C48/80-stimulated Ca^2+^ influx.

We further investigated the expression of Ca^2+^ influx–related proteins (TRPC1, Orai1, STIM1, *PLC-γ,* and IP3R). Based upon western blotting results, the expression levels of TRPC1, Orai1, PLCγ, and IP3R were significantly decreased by whole QE and QE albumen (*p* < 0.05, Fig. 5b-g), resulting in almost 45–75% fold reduction in protein expression. In addition, even though the inhibition effects of QE on these protein expressions were not as strong as QE albumen (*p* < 0.05), which resulted only in 20–40% fold reduction in protein expression, QE yolk also showed significant inhibition effects on the expression of TRPC1, Orai1, and IP3R, as compared to control group (*p* < 0.05; Fig. 5c-d;g). However, QE yolk showed no modulation effect on regulating STIM-1 and PLCγ protein expressions (*p* > 0.05; Fig. 5e-f). We then confirmed that QE, particularly its albumin, had a more significant effect on the depletion of ER calcium store, as compared to QE yolk, by stabilizing mast cells through significantly suppressing the Ca^2+^ influx due to the lower expression of calcium channel proteins (TRPC1, Orai1, STIM1, PLC-γ, and IP3R).

### The effect of QE on the PAR-2-mediated MAPK and NF-kB cell signaling pathways of HMC-1 cells

**Fig. 6:**
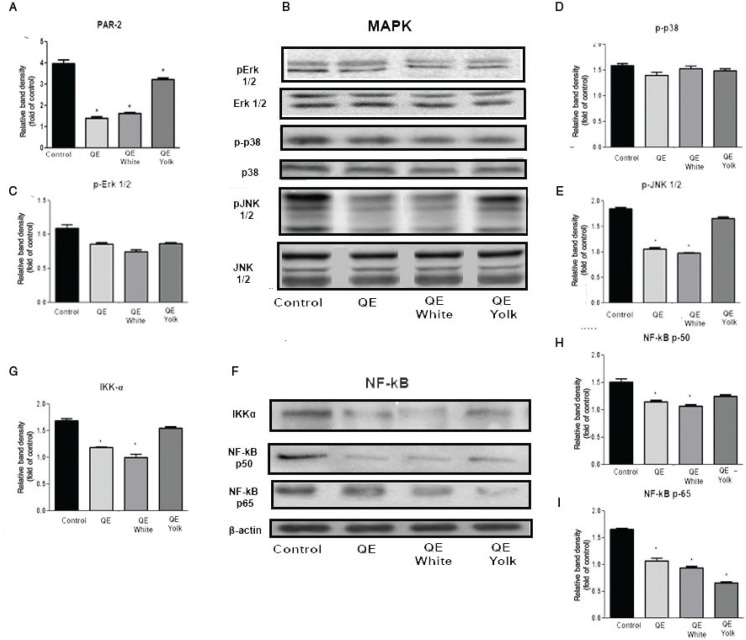
The effect of QE on the PAR-2-mediated MAPK and NF-kB cell signaling pathways of HMC-1 cells. (a) mRNA fold induction of PAR-2. (b) Western blotting of MAPK signaling. MAPK fold activation data analysis: (c) pERK1/2, (d) p-p38 and (e) p-JNK1/2. (f) Western blotting of NF-kB signaling. NF-kB fold activation data analysis: (g) IKK-α, (h) NF-kB p50, and (i) NF-kB p65. Results are expressed as mean ± SEM. *p < 0.05 as compared to the control group.

It has been reported that QE plays an important role in the treatment of allergy disease by acting as a PAR-2 inhibitor which is able to enhance mast cell stabilization (6). In addition, our recent study has also indicated the significant PAR-2 activation in peanut allergy–induced EoE-like disease murine model and oral QE treatment was able to diminish the elevation of PAR-2 expression. Then, in this experiment, the effect of QE treatments on expression of PAR2 was examined by RT-PCR. Indeed, we found a similar trend with our previous study. As shown in Fig. 6a, PAR-2 expression was significantly elevated in the HMC-1 control group and whole QE and QE albumen treatments were able to significantly inhibit PAR-2 expression (*p* < 0.05), while QE yolk inhibition was weaker as compared to other QE treatments (*p* < 0.05).

Because the activation of MAPK and NF-κB is critically required for the transcriptional regulation of PAR-2-mediated allergic response (24), we further investigated whether the inhibition of allergic responses by QE are mediated through the MAPK and nuclear translocation of NF-κB pathway in C48/80-stimulated HMC-1 by western blot analysis. As shown in Fig. 6b-e, C48/80 markedly stimulated the phosphorylation of ERK 1/2, JNK 1/2, and p38 MAPK as well as IKKα, nuclear translocations of p50 and p65 subunit of NF-κB in HMC-1 (Fig. 6f-i). QE pretreatment, particularly QE albumen, significantly suppressed the C48/80-induced activation of JNK MAPK (*p* < 0.05, Fig. 6e), resulting in 47.10 ± 1.39% fold protein reduction, but did not affect the phosphorylation of ERK or p38 (*p* > 0.05; Fig. 6c-d), and QE yolk did not show any inhibition effect (*p* > 0.05; Fig. 6b-e). Furthermore, IKKα, NF-kB p50, and p65 were also affected by C48/80 (Fig. 6f-i). Similar to the result of MAPK pathway, QE, particularly QE albumen, significantly suppressed the activation of IKK-α and NF-kB p50, resulting in 54.29 ± 1.21% and 16.67 ± 1.14% fold protein reduction (*p* < 0.05; Fig. 6g-h), while QE yolk did not show any inhibition effect (*p* > 0.05; Fig. 6g-h). Surprisingly, all QE treatment groups showed a significant inhibition effect on modulating NF-kB p65 activation as compared to control group (*p* < 0.05; Fig. 6i), resulting in almost 40–60% fold protein reduction where QE yolk appeared as the most effective treatment among the others which resulted in 58.83 ± 1.15% fold protein reduction (*p* < 0.05; Fig. 6i). All the results indicated that QE, particularly its albumin, played an important role in the modulation of PAR-2-mediated MAPK and NF-kB translocation signaling pathways.

## Discussion

QE has been shown to have diverse biological activities, such as anti-allergic, anti-inflammatory, and anti-cancer activities (4–10, 24, 25). Several clinical studies have indicated that daily QE oral treatment could attenuate allergic asthmatic and rhinitis symptoms (5, 8, 9). In addition, our recent study found that QE was able to modulate the inflammatory response of food allergy–induced EoE-like disease by modulating PAR-2 transduction pathway in peanut-sensitized mice (10). Even though we have reported that QE inhibited the activation of PAR-2 in EoE murine model which represented delayed allergic response, the suppressive effect of QE on modulating mast cell degranulation in immediate allergic response is not yet well defined. So far, *in vitro* studies have just reported the role of QE on modulating the activation of basophils, neutrophils, and eosinophils. Therefore, to verify this, we firstly confirm the role of QE as ‘mast cell stabilizer’ *in vivo* using a IgE-mediated immediate allergic response in PCA mice model by which the report in accordance to this appears to be minimal. Then, we used HMC-1 mast cell lines to study the suppressive effect of QE on mast cell degranulation.

**Fig. 7:**
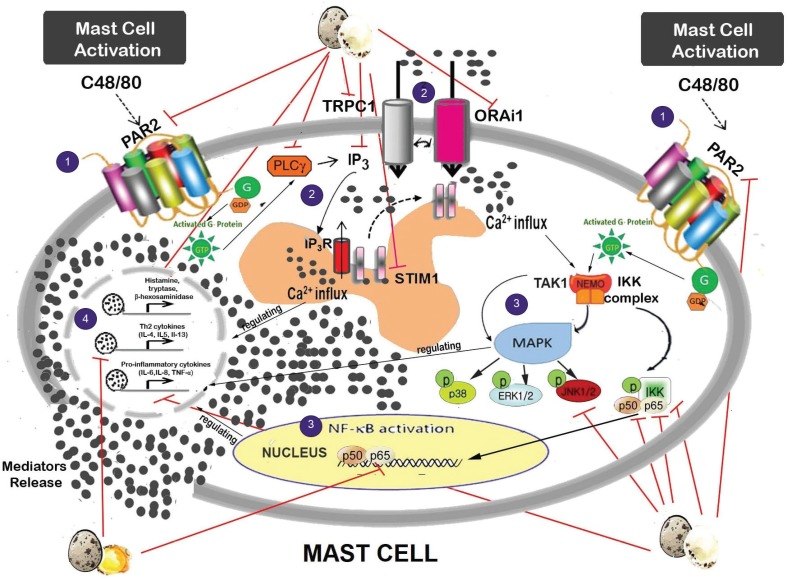
Schematic diagram of the suppressive role QE on modulating mast cell activation. QE acts as a ‘mast cell stabilizer’ to reduce mediator release through 1) modulating PAR-2 activation; (2) induces the downregulation of calcium channel proteins (TRPC1, Orai1, STIM1, PLC-γ, and IP3R); (3) leads to the reduction of phosphorylation JNK, NF-kBp50, and p65, as well as IKK-α contributed in MAPK and NF-kB signaling pathways related to mast cell degranulation stimulated by antigen; and thus (4) promotes the decrease level of secretion mediators (β-hexosaminidase, histamine, tryptase) including Th2 (IL-4, IL-5, and IL-13) and pro-inflammatory related cytokines (IL-6, IL-8, and TNF-α) release.

As summarized in Fig. 7, this present study found that QE possessed an anti-allergic activity through suppressing mast cell activation. It has been well-established that allergic mediator releases, like histamine, tryptase, Th2, and pro-inflammatory related cytokines, are closely associated with various allergic and inflammatory diseases. Thus, the inhibition of allergic mediators’ generation by mast cells is an important therapeutic strategy in the context of allergic-inflammatory disease. In this present finding, we observed that QE albumen played the most effective role as compared to QE yolk in modulating mast cell degranulation by suppressing the release of β-hexosaminidase, histamine, and tryptase, as well as pro-inflammatory cytokines (IL-6, IL-8, and TNF-α) and upregulating the release of IL-10 in a dose-dependent manner, by which data showed that the lowest concentration QE albumen (10 μg/mL) already had significant inhibitory effect on modulating these mediators. In addition, even though QE yolk also showed significant therapeutic effect to modulate these mediators, these modulation effects were not as strong as QE albumen, by which its significant inhibition effects started in concentration of 50 μg/mL. Interestingly, although QE albumen in lower concentration also showed significant inhibitory effects on mast cells released Th2 (IL-4, IL-5, IL-13) and pro-inflammatory cytokines (IL-6, IL-8), QE yolk showed a greater significant inhibition effect as compared to QE albumen on modulating those allergic related cytokines, even though its inhibition effect was also in a reverse dose-dependent manner.

The augmentation of Th2 cytokines in a higher concentration of QE was not surprising as it is largely known that QE itself contained many described egg allergens which also may act on immune pathway regulation to provide benefit in the occurrence of allergy reactions. Besides, the effective effect of QE yolk on modulating Th2 cytokines likely due to its high nutrient contents which likely also play an important role as anti-allergic agents (26). In contrast, study using chicken egg albumen to treat trimellitic anhydride (TMA) induced allergy murine model supported our finding. This study reported that TMA-sensitized mice treated with chicken egg albumen did not show any increase of IgE specific level, but the consumption of chicken egg albumen by TMA-treated mice could not modulate mice Th2 immune responses (27). However, it is still difficult to take the conclusion by comparing the effect of chicken egg and QE albumen only. Even though a proteomic study has reported that QE has other special protein fractions which may play a beneficial role as an anti-allergic agent (28), another proteomic study using egg albumen of six different bird species (chicken, duck, goose, turkey, quail, and pigeon) discovered that the content of QE albumin major protein allergens (ovalbumin, ovomucoid, ovotransferrin, lysozyme) were similar to other bird egg albumin species (29). Therefore, we considered no modulation effect on Th2 cytokines in cells treated with high QE albumen concentrations was likely due to the presence of these major protein allergens. However, detailed anti-allergic activity of QE still needs to be elucidated.

Following mast cell activation, the production of IP_3_ allows calcium influx through both non-calcium selective canonical transient receptor channel family (TRPC) channels, and/or the highly calcium selective store-operated calcium entry channels (Orai). However, IP_3_ in initiating internal calcium store depletion acts indirectly, which is identified by the ER resident protein STIM1 (30, 31), that in turn causes activation of Orai channels (32–34). The increased level of intracellular Ca^2+^ concentration was significantly reduced after QE albumen treatment, which suggested that QE also plays a stabilizing role on mast cells by inhibiting the extracellular Ca2+ influx process. The decreased expression of Orai1, STIM1 and TRPC1 in protein levels indicate that GA might suppress the Ca2+-dependent degranulation due to the lower expression of these calcium channel proteins. Besides, the downregulation of IP3R protein expression in mast cell treated with QE albumen indicated that QE also has an effect on the depletion of ER Ca^2+^ store. A marked upregulation of the calcium channel proteins activation provokes the activation of PAR-2. Its activation, through coupling with G proteins, induces a variety of signaling cascades including PLCγ activation, which thus evokes [Ca^2+^]_i_ rise and then triggers both MAPK and NF-kB signaling pathways in mast cells, leading to the release of mediators. In this study, we also found that QE albumen effectively suppressed the activation of PLC-γ in protein level. Next, we also found that QE also significantly suppressed the phosphorylation of JNK, p50, p65, as well as IKKα which represented MAPK and NF-kB signaling pathways, respectively. In addition, even though QE yolk’s role in modulating mediators release is not as strong as QE albumen, QE yolk shows a significant effect on modulating NF-kB downstream signaling pathway through effectively modulating the phosphorylation of p65. This finding is in line with our previous study which shows that QE can block the activation of PAR-2 through inhibiting the phosphorylation of NF-kB p65 in food allergy–induced EoE mice model. Taken together with our previous results (10), the anti-allergic inflammatory activity of QE appears to be due to the suppressions of the secretions of allergic mediators and intracellular Ca^2+^ influx generation through the inhibition of PAR-2 downstream signaling transduction pathway.

In conclusion, as confirmed by passive cutaneous anaphylaxis and HMC-1 cell-based immunology assay, QE albumen and QE yolk may work together through exerting anti-allergy activity and can be used as a potential anti-allergic nutrient in the future.
